# Assessment of Left Atrial Fibrosis With Cardiac MRI

**DOI:** 10.1002/jmri.70103

**Published:** 2025-08-26

**Authors:** Monique Bernard

**Affiliations:** ^1^ Aix‐Marseille Univ, CNRS, CRMBM Marseille France

**Keywords:** 3D late gadolinium‐enhanced sequence (3D‐LGE), atrial fibrillation, cardiac MRI, fibrosis

1

Atrial fibrillation (AF) is the most common sustained arrhythmia. AF is associated with an increased risk of thromboembolic stroke, heart failure with both an increased morbidity and mortality, and increasing worldwide prevalence [1]. AF is associated with structural, electrical, and functional alterations of the left atrium [2]. Left atrial (LA) fibrosis is a central component of structural remodeling and plays a major role in the pathogenesis and progression of atrial fibrillation [3]. LA fibrosis burden is used to assign the degree of LA fibrosis into 4 quartiles (Utah stages) [4]. Radiofrequency catheter ablation via pulmonary vein isolation (PVI) or pharmaceutical therapy is commonly used to treat AF depending on the fibrosis stage [5]. Assessing accurately the extent of fibrosis is therefore important for treatment stratification [6].

While histopathology is the reference standard, atrial fibrosis can be assessed with late gadolinium enhancement (LGE) MRI or with invasive electroanatomic voltage mapping (EAVM) [7]. Several studies have compared LGE MRI and EAVM, and the level of agreement has been shown to be dependent on acquisition and post‐processing parameters [8] or on the level of atrial cardiomyopathy severity [7] emphasizing the need for parameter harmonization.

3D late gadolinium‐enhanced (3D‐LGE) sequence provides high‐resolution images to identify LA fibrosis. Different image processing and fibrosis quantification methods can be used to quantify LA fibrosis using 3D‐LGE, based on open‐source or commercial software packages [6, 9, 10]. After performing segmentation or using computational left atria models [10] the most commonly used approaches are based on thresholding, and two of these methods are mostly used [9]. The SD method defines a number of standard deviations (SDs) above a reference value, usually the mean signal intensity for normal myocardium or mean signal intensity of the LA blood pool. A threshold of two or three SDs above the mean blood pool signal intensity is generally proposed using the SD method [9]. On the other hand, the image intensity ratio (IIR) method normalizes the signal intensity of the LA wall to the mean blood pool signal intensity. Thresholds of 0.97, 1.2, or 1.32 are generally used to indicate fibrosis [9, 10].

Studies show that atrial fibrotic burden is dependent on the post‐processing method used as well as on the defined threshold [7, 9]. Comparison of different threshold values and various post‐processing software as well as validation in large cohorts and multicenter studies and validation against histopathological data or mapping methods are needed to harmonize practices for LA fibrosis quantification using LGE.
